# Folate Reverses NF-κB p65/Rela/IL-6 Level Induced by Hyperhomocysteinemia in Spontaneously Hypertensive Rats

**DOI:** 10.3389/fphar.2021.651582

**Published:** 2021-09-16

**Authors:** Lihua Zhang, Zhongliang Li, Changcheng Xing, Ning Gao, Rui Xu

**Affiliations:** ^1^ Cheeloo College of Medicine, Shandong Qianfoshan Hospital, Shandong University, Jinan, China; ^2^ Department of Medicine, Jinan Maternity and Child Care Hospital Affiliated to Shandong First Medical University, Jinan, China; ^3^ Department of Women Healthcare, Jinan Maternity and Child Care Hospital Affiliated to Shandong First Medical University, Jinan, China; ^4^ Department of Cardiology, The First Affiliated Hospital of Shandong First Medical University, Jinan, China

**Keywords:** hypertension, hyperhomocysteinemia, folate, oxidative stress, arterial inflammation

## Abstract

Hyperhomocysteinemia (HHcy) is derived from the abnormal metabolism of homocysteine (Hcy) and is related to metabolic-related diseases. In addition, HHcy combined with hypertension increases the risk of cardiovascular diseases (CVD). However, the mechanism of HHcy aggravating hypertensive arterial damage and the efficacy of folate (FA) as a beneficial supplement have not been fully elucidated. In this study, we established a rat HHcy model and a hypertension combined with HHcy model. Rat tail artery blood pressure (BP), plasma Hcy, serum superoxide dismutase (SOD), and malondialdehyde (MDA) were measured. Rat thoracic aorta was for pathological analysis after 12 weeks of the experiment. The relative expression levels of oxidative stress and immune/inflammation in rat arterial tissues were detected by quantitative real-time polymerase chain reaction (qRT-PCR) and western blotting. The results demonstrated that the relative expression levels of oxidative stress and immune/inflammation were the highest in the hypertension combined with HHcy group, followed by the hypertension group. Compared with the hypertension group, the hypertension combined with HHcy group up-regulated the expression levels of interleukin-6 (IL-6) and nuclear factor-κ-gene binding (NF-κB) p65/Rela, but not NADPH oxidase (Nox). Furthermore, folate inhibited the expression of IL-6 and NF-κB p65/Rela, reduced the levels of MDA and HHcy, but significantly increased the SOD level. In conclusion, HHcy synergistically aggravated the arterial damage factor of hypertension through immune/inflammatory response. However, folate demonstrated anti-inflammatory properties and reversed the NF-κB p65/Rela/IL-6 level induced by HHcy in hypertensive rats.

## Introduction

Epidemiological studies have found that the incidence of hypertension combined with hyperhomocysteinemia (HHcy) is relatively high in China (Li et al., 2007). In addition, HHcy also significantly increases the incidence of cardiovascular disease (CVD) (Chen et al., 1963). The special feature of hypertension combined with HHcy is the combination of HHcy, which is the abnormal accumulation of homocysteine (Hcy) in the body. Hcy is a metabolic-intermediate product of dietary methionine after demethylation and is also a pivotal molecule connecting tetrahydrofolate metabolism and trans-sulfur metabolism (Henrieta et al., 2016; Weber et al., 2016). The main causes of HHcy (Fowler, 2005; Asfar and Safar, 2007; Wierzbicki, 2007; Humphrey et al., 2008) include the metabolic enzyme deficiency (catalytic enzyme involved in the Hcy metabolic pathway), cofactor deficiency (vitamin B), excessive intake of methionine, certain diseases, some drugs and the excretion dysfunction of the kidney. Therefore some scholars propose that HHcy is a marker (Fu et al., 2017) of abnormal methyl metabolism (including methionine metabolism and folate metabolism) and/or abnormal transsulfur metabolism in the body. Another statement indicates that the unique thiol structure of HHcy/Hcy has molecular toxicity and is the cause of CVD (Gurda et al., 2015).

Studies have found that HHcy is mostly accompanied by an increase in reactive oxygen species (ROS) and inflammation (Hu et al., 2018; Yunkai et al., 2018), regardless of whether HHcy is a marker of abnormal metabolism in the body or an independent pathogenic factor for CVD. Additionally, the levels of oxidative stress and immune/inflammation are also increased in patients with hypertension (Guzik and Touyz, 2017). It is known that NADPH oxidase (Nox) enzyme is an important member of integrated stress response in the body and is also the main source of ROS (Cifuentes-Pagano et al., 2012; Chuong Nguyen et al., 2015). In addition, the Nox family is highly expressed in vascular tissues, among which Nox4 is more expressed in endothelial cells (Bedard and Krause, 2007). Furthermore, the NF-κB family is widely involved in tissue cell metabolism and is also a significant immune/inflammation response molecule (Polesso et al., 2017). Its activating molecule NF-κB p65/Rela can induce inflammatory cytokines, such as tumour necrosis factor-alpha (TNF-a) and interleukin-6 (IL-6) (Proto et al., 2015), while inactive molecule NF-κB2 is the opposite. However, the mechanism of HHcy promoting hypertensive arterial damage and the relationship between hypertension combined with HHcy and the Nox/NF-κB pathway molecules are not fully understood.

Epidemiological studies found (Holt et al., 2009) that a diet high in fruit and vegetables, rich in folate, is associated with lower levels of markers of inflammation and oxidative stress. Folate is one of the essential members of group B vitamins and provides a significant one-carbon unit for the methylation modification required by the body’s metabolism. Moreover, folate also promotes the conversion of Hcy to methionine to significantly reduce the concentration of HHcy (Chan et al., 2017; Vezzoli et al., 2020; Zhao et al., 2020). Therefore, in China, folate has been used as an important pharmaceutical ingredient against hypertension combined with HHcy (Li et al., 2007). Besides, folate is also a powerful antioxidant (Jiang et al., 2010) and is closely associated with activated immune cells that highly express folate receptors (Cifuentes-Pagano et al., 2012; Chuong Nguyen et al., 2015). However, the precise mechanism underlying folate against arterial injury of hypertension combined with HHcy has not been fully elucidated. In particular, it is still unclear whether folate is involved in the mechanisms of anti-inflammation and immune regulation.

In this study, we constructed three models: HHcy rats model (HHcy group), spontaneously hypertensive rats (SHR) combined with HHcy model (HHcy + SHR group), and folate (FA) intervention SHR combined with HHcy model (HHcy + SHR + FA group). We observed the pathology of rat artery and compared the oxidative stress and immune-inflammatory factors, in order to explore the pharmacological effects of folate on arterial damage exacerbated by HHcy and the underlying cellular and molecular mechanisms of HHcy and folate.

## Material and Methods

### Animals

Sixteen male Wistar-Kyoto rats (WKY) and 24 male spontaneously hypertensive rats (SHR) (250–270 g, 12 weeks old) were obtained from the Beijing Vital River Laboratory Animal Center (Beijing, China). They were maintained on a 12:12 h light/dark cycle (lights on 08:00–20:00) in an air-conditioned constant temperature (22 ± 2°C) colony room, with free access to water and food. Animal care and experimental protocol for this study were approved by the Committee on the Use of Live Animals in Teaching and Research of Qianfoshan Hospital. The Laboratory Animal Unit of Qianfoshan Hospital was fully accredited by the Association for Assessment and Accreditation for Laboratory Animal Care (AAALAC International).

### Experimental Grouping

**FIGURE 1:**
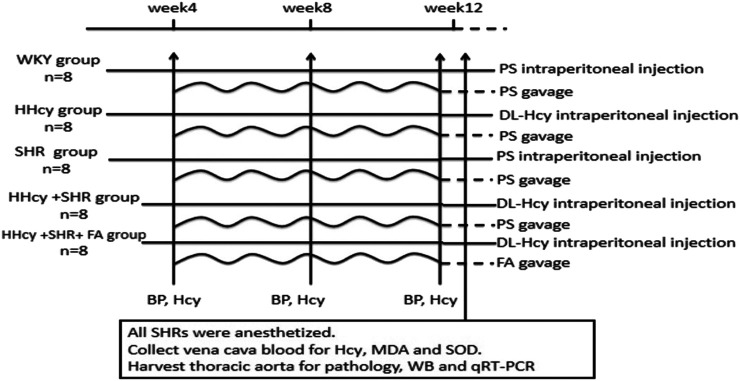
Diagram of the study design. The horizontal lines represent the intraperitoneal injection of different drug. The wavy lines represent the gavage of different drug. The arrows refer to the intervention points of different experimental operations. WKY, Wistar-Kyoto; HHcy, hyperhomocysteinemia; SHR, spontaneously hypertensive rat; FA, folate; PS, physiological saline; Hcy, homocysteine; MDA, malondialdehyde; SOD, superoxide dismutase; WB, western blotting; qRT-PCR, quantitative real-time polymerase chain reaction.

The sixteen male WKYs were randomly distributed into two experimental groups: WKY group and HHcy group (n = 8/group); the 24 male SHRs were randomly distributed into three experimental groups: SHR group, HHcy + SHR group and HHcy + SHR + FA group (n = 8/group). The rats in the WKY group and the SHR group were administered physiological saline (PS, 5 ml/kg, twice a day) intraperitoneally for 12 weeks. Additionally, the rats in the HHcy group, the HHcy + SHR group and the HHcy + SHR + FA group were injected intraperitoneally with 2% DL-Hcy (5 ml/kg, twice a day, H4628, Sigma-Aldrich, St. Louis, United States) for 12 weeks. During the last 8 weeks of the experiment, the HHcy + SHR + FA group was given folate (0.4 mg/kg/d, F7876, Sigma) by gavage, and the other four groups were given gavage of the same amount of PS. The folate was freshly dissolved in 0.5 ml PS immediately before gavage (Figure 1).

### Blood Pressure Measurement

Systolic blood pressure (SBP) and diastolic blood pressure (DBP) of rat tail artery were monitored at the same time of day with a noninvasive BP measurement system (Beijing Ruolong Biotechnology Company, BP-2010A) under rat conscious state. All rats were tested for BP at least every 4 weeks in the 12-week experiment. For each BP test, each rat was subjected to at least three consecutive BP measurements, and an average BP value was taken as the final BP of each rat.

### Specimen Preparation

Rats were anesthetized by intraperitoneal injection of sodium pentobarbital (50 mg/kg, i. p.), and then blood samples were collected from vena cava to measure plasma Hcy, serum malondialdehyde (MDA), and superoxide dismutase (SOD). Before regaining sensation, the rats were euthanized by bleeding and then their thoracic aorta was harvested. Each thoracic aorta was divided into two parts: one part was prepared by standard methods for pathological analysis; the other part was immediately frozen in liquid nitrogen, and then was stored at −80°C to measure protein levels by western blotting and mRNA levels by quantitative real-time polymerase chain reaction (qRT-PCR).

### Measurement of Hcy, MDA, and SOD

All rat blood samples were sent to the laboratory of Qianfoshan Hospital for measurement. The concentration of plasma Hcy was measured by using a Cobas8000 automatic biochemistry analyzer (Roche, Switzerland). The activity of serum SOD and the level of serum MDA were determined by using commercial kits (Jiancheng Institute of Biological Technology, Nanjing, Jiangsu, China) according to the manufacturer’s instructions.

### Histopathological Analysis

Thoracic aortas slices (5 μm thick) were deparaffinized and subjected to hematoxylin-eosin (HE) staining (Service Biological Technology Co., Ltd., Wuhan, China). Images of three microscopic fields in each slice were captured (magnification, ×400) under an inverted fluorescence microscope (Olympus, Tokyo, Japan), and the relative count of vascular smooth muscle cells (VSMCs) was measured by Image-Pro Plus 6.0 software, independent of the orientation, form or size of cell nucleus.

Thoracic aortas slices (5 μm thick) were deparaffinized and subjected to Masson staining (Service Biological Technology Co., Ltd., Wuhan, China). Images of three microscopic fields in each slice were captured (magnification, ×400) under an inverted fluorescence microscope (Olympus, Tokyo, Japan), and collagen deposition in the vascular wall was analyzed using Image-Pro Plus 6.0 (Media Cybernetics, Inc., Rockville, MD, United States). The ratio of collagen (blue) to the fixed area of the thoracic aorta was calculated as the result of semi-quantitative analysis of collagen deposition.

### qRT-PCR

Total RNA was extracted from the homogenate of fresh-frozen thoracic aorta without adipose tissue using TRIzol reagents (Invitrogen, 15596026). Afterwards, the RNA was quantified spectrophotometrically (Spectrophotometer, Merinton, SMA4000) and was reverse transcribed into cDNA with RT reagent kit with gDNA Eraser (Takara, RR047A) for qRT-PCR. The primer sequences of IL-6, TNF-α, NF-κB p65/Rela, NF-κB2, Nox2, and Nox4 were used to determine gene expression using TB Green Premix Ex TaqⅡ (Takara, RR820A) in a real-time PCR machine (ABI ViiA 7, Applied Biosystems, Foster City, CA). The program was run with reaction cycling of initial denaturing (95°C, 30 s), followed by 40 cycles of denaturing (95°C, 5 s) and extension (60°C, 30 s). Additionally, a melting curve was run to confirm specificity of PCR products. At last, gene expression levels were normalized to GAPDH expression levels, and the relative quantity of mRNA expression was calculated according to the cycle threshold (2^−△△Ct^) method. The primer sequences of target genes for amplification are listed in Table 1.

**TABLE 1:** Primer sequences for quantitative real-time polymerase chain reaction.

Name	Sequence -F	Sequence -R
TNF-α	GGC​GTG​TTC​ATC​CGT​TCT​C	CTT​CAG​CGT​CTC​GTG​TGT​TTC​T
IL-6	ATT​GTA​TGA​ACA​GCG​ATG​ATG​CAC	CCA​GGT​AGA​AAC​GGA​ACT​CCA​GA
NOX2	CCT​GGA​GAC​CCA​GAT​GCA​AGA	CGT​GGT​GCA​CAG​CAA​AGT​GA
NOX4	ACT​GGT​GAA​GAT​TTG​CCT​GGA​AG	CAC​AGT​ATA​GGC​ACA​AAG​GTC​CAG​A
NF-κB p65/Rela	ATC​CCT​GCT​TCC​CCT​TTC​TC	CTG​TCT​TAT​GGC​TGA​GGT​CTG​GT
NF-κB _2_	CTG​ATG​GCA​CAG​GAC​GAG​AA	TGG​GCT​ATC​TGC​TCA​ATG​ACA​C

### Western Blotting

Total proteins were extracted from the homogenate of fresh-frozen thoracic aorta without adipose tissue by using the Protein Extraction Kit (invent, SA-03-BV). Protein samples (30 μg per lane) were separated by SDS-PAGE and were transferred to PVDF membrane. Afterwards, the PVDF membrane was blocked with 5% milk in Tris-buffered saline Tween and incubated with primary antibodies (Anti-IL-6, PTG, 21865-1-AP; Anti-TNF-α, PTG, 17590-1-AP; Anti-NOX2, Abcam, ab129068) overnight at 4°C. Then the PVDF membranes were washed with Tris-buffered saline Tween solution and incubated with horseradish peroxidase-conjugated second antibody for 1 h. The ChemiDoc™ Touch Gel imaging system (Bio-Rad, Hercules, CA, United States) was used to visualize immunoreactivity with a chemiluminescent HRP substrate (Vazyme, Nanjing, Jiangsu, China). The band intensities were determined using Image Lab software and expressed relative to GAPDH.

### Statistical Analysis

Statistical analysis was conducted using one-way ANOVA followed by the LSD test. Results were expressed as means ± SD. All statistical analyses were performed using SPSS software version 13.0. *p* < 0.05 was considered statistically significant.

## Results

### Comparison on Hcy Between Groups

**FIGURE 2:**
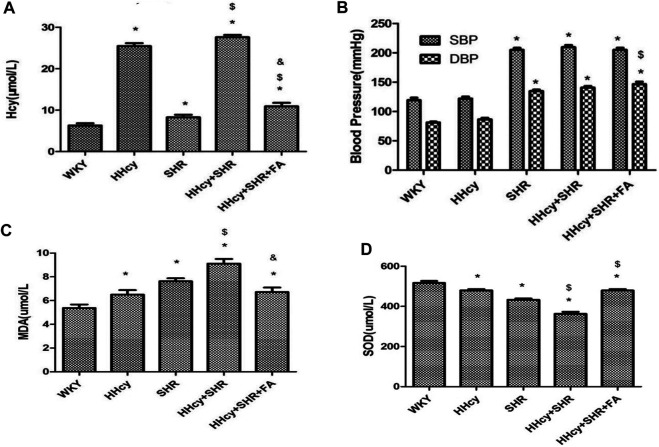
The levels of homocysteine (Hcy), superoxide dismutase (SOD), malondialdehyde (MDA), systolic blood pressure (SBP), and diastolic blood pressure (DBP) in Wistar-Kyoto (WKY) group, hyperhomocysteinemia (HHcy) group, spontaneously hypertensive rat (SHR) group, HHcy + SHR group, and HHcy + SHR + folate (FA) group. **(A)** The levels of Hcy. **(B)** The levels of SBP and DBP. **(C)** The levels of MDA. **(D)** The levels of SOD. Values represent means ± SD (*p < 0.05 vs. the WKY group, ^$^ *p* < 0.05 vs. the SHR group, ^&^ *p* < 0.05 vs. the HHcy + SHR group, n = 8).

After intraperitoneal injection of DL-Hcy, rat plasma Hcy levels were significantly increased in the HHcy group than the WKY group (HHcy group, 25.48 ± 2.01 μmol/L vs. WKY group, 6.30 ± 1.47 μmol/L; *p* < 0.05, n = 8). Similarly, the levels of serum Hcy were also significantly increased in the HHcy + SHR group than the SHR group (HHcy + SHR group, 27.61 ± 1.53 μmol/L vs. SHR group, 8.26 ± 1.77 μmol/L; *p* < 0.05, n = 8). The Hcy levels of both the HHcy group and the HHcy + SHR group were more than 15 μmol/L, which fully complies with the HHcy standard (Hu and Xu, 2009). After folate treatment, the plasma Hcy levels were significantly lower in the HHcy + SHR + FA group compared with the HHcy + SHR group (HHcy + SHR + FA group, 10.93 ± 2.34 μmol/L vs. HHcy + SHR group, 27.61 ± 1.53 μmol/L; *p* < 0.05, n = 8). However, the Hcy levels of the HHcy + SHR + FA group were still significantly higher than those of the SHR group (HHcy + SHR + FA group, 10.93 ± 2.34 μmol/L vs. SHR group, 8.26 ± 1.77 μmol/L; *p* = 0.007, n = 8). Moreover, the Hcy levels of the SHR group were also higher than those of the WKY group (SHR group, 8.26 ± 1.77 μmol/L vs. WKY group, 6.30 ± 1.47 μmol/L; *p* = 0.041, n = 8). (Figure 2A; Table 2).

**TABLE 2:** The levels of Hcy, MDA, SOD, SBP, and DBP in five groups.

Group	WKY (n = 8)	HHcy (n = 8)	SHR (n = 8)	HHcy + SHR (n = 8)	HHcy + SHR + FA (n = 8)
Hcy (μmol/L)	6.30 ± 1.47	25.48 ± 2.01*	8.26 ± 1.77*	27.61 ± 1.53*^$^	10.93 ± 2.34*^$&^
MDA (nmol/mL)	5.37 ± 0.85	6.50 ± 1.07*	7.63 ± 0.70*	9.11 ± 1.12*^$^	6.72 ± 1.05*^a&^
SOD (U/mL)	516.85 ± 28.69	479.64 ± 18.38*	432.50 ± 19.73*	362.73 ± 30.23*^$^	479.04 ± 20.62*^$a&^
SBP (mmHg)	119.25 ± 11.85	121.88 ± 9.89	205.13 ± 9.54*	209.75 ± 9.81*	205.25 ± 9.45*
DBP (mmHg)	81.13 ± 4.32	86.63 ± 6.80	134.88 ± 6.73*	140.63 ± 7.39*	146.63 ± 11.50*^$^

Hcy, homocysteine; MDA, malondialdehyde; SOD, superoxide dismutase; SBP, systolic blood pressure; DBP, diastolic blood pressure; HHcy, hyperhomocysteinemia; FA, folate. Values represent means ± SD (*p < 0.05 vs. the WKY group; ^$^ *p* < 0.05 vs. the SHR group; ^&^ *p* < 0.05 vs. the HHcy + SHR group; n = 8).

### Comparison on BP Between Groups

After intraperitoneal injection of DL-Hcy, the SBP, and DBP of HHcy rats were slightly higher than those of the WKYs, but the differences between the two groups were not statistically significant (HHcy group: SBP, 121.88 ± 9.89 mmHg; DBP, 86.63 ± 6.80 mmHg vs. WKY group: SBP, 119.25 ± 11.85 mmHg; DBP, 81.13 ± 4.32 mmHg; *p* > 0.05, n = 8). A similar result also existed in the HHcy + SHR group compared with the SHR group (HHcy + SHR group: SBP, 209.75 ± 9.81 mmHg; DBP, 140.63 ± 7.39 mmHg vs. SHR group: SBP, 205.13 ± 9.54 mmHg; DBP, 134.88 ± 6.73 mmHg; *p* > 0.05, n = 8). After folate treatment, there was no significant BP change between the HHcy + SHR + FA group and the HHcy + SHR group. In summary, the above results showed that neither HHcy nor folate had a significant effect on BP changes (Figure 2B; Table 2).

### Comparison on Oxidative Stress Indicators in Peripheral Blood

After intraperitoneal injection of DL-Hcy, the levels of serum MDA, as a product of ROS oxidized lipids, were significantly increased in the HHcy group rats compared with the WKYs (HHcy group, 6.50 ± 1.07 nmol/ml vs. WKY group, 5.37 ± 0.85 nmol/ml; *p* = 0.026, n = 8). The same result also appeared in the HHcy + SHR group compared with the SHR group (HHcy + SHR group, 9.11 ± 1.12 nmol/ml vs. SHR group, 7.63 ± 0.70 nmol/ml; *p* = 0.004, n = 8). After folate treatment, rat serum MDA levels were significantly lower in the HHcy + SHR + FA group than the HHcy + SHR group (HHcy + SHR + FA group, 6.72 ± 1.05 nmol/ml vs. HHcy + SHR group, 9.11 ± 1.12 nmol/ml; *p* < 0.05, n = 8). However, there was no significant difference between the HHcy + SHR + FA group and the SHR group (HHcy + SHR + FA group, 6.72 ± 1.05 nmol/ml vs. SHR group, 7.63 ± 0.70 nmol/ml; *p* = 0.069, n = 8). (Figure 2C; Table 2).

After intraperitoneal injection of DL-Hcy, the levels of serum SOD, as a molecule against ROS, were significantly lower in the HHcy rats compared with the WKYs (HHcy, 479.64 ± 18.38 U/mL vs. WKY, 516.85 ± 28.69 U/mL; *p* = 0.004, n = 8). The same result also appeared in the HHcy + SHR group compared with the SHR group (HHcy + SHR group, 362.73 ± 30.23 U/mL vs. SHR group, 432.50 ± 19.73 U/mL; *p* < 0.05, n = 8). After folate treatment, rat serum SOD levels were significantly higher in the HHcy + SHR + FA group than the HHcy + SHR group (HHcy + SHR + FA group, 479.04 ± 20.62 U/mL vs. HHcy + SHR group, 362.73 ± 30.23 U/mL; *p* < 0.05, n = 8). Moreover, the SOD levels of the HHcy + SHR + FA group were still significantly higher than those of the SHR group (HHcy + SHR + FA group, 479.04 ± 20.62 U/mL vs. SHR group, 432.50 ± 19.73 U/mL; *p* < 0.05, n = 8) (Figure 2D; Table 2).

In summary, the HHcy + SHR group significantly increased the oxidative stress level (increasing MDA and decreasing SOD) in the blood circulation, followed by the SHR group and the HHcy group. Folate therapy reduced the level of oxidative stress in peripheral blood, especially by significantly increasing the level of anti-oxidative stress molecule SOD.

### Comparison on Aortic Pathology

**FIGURE 3:**
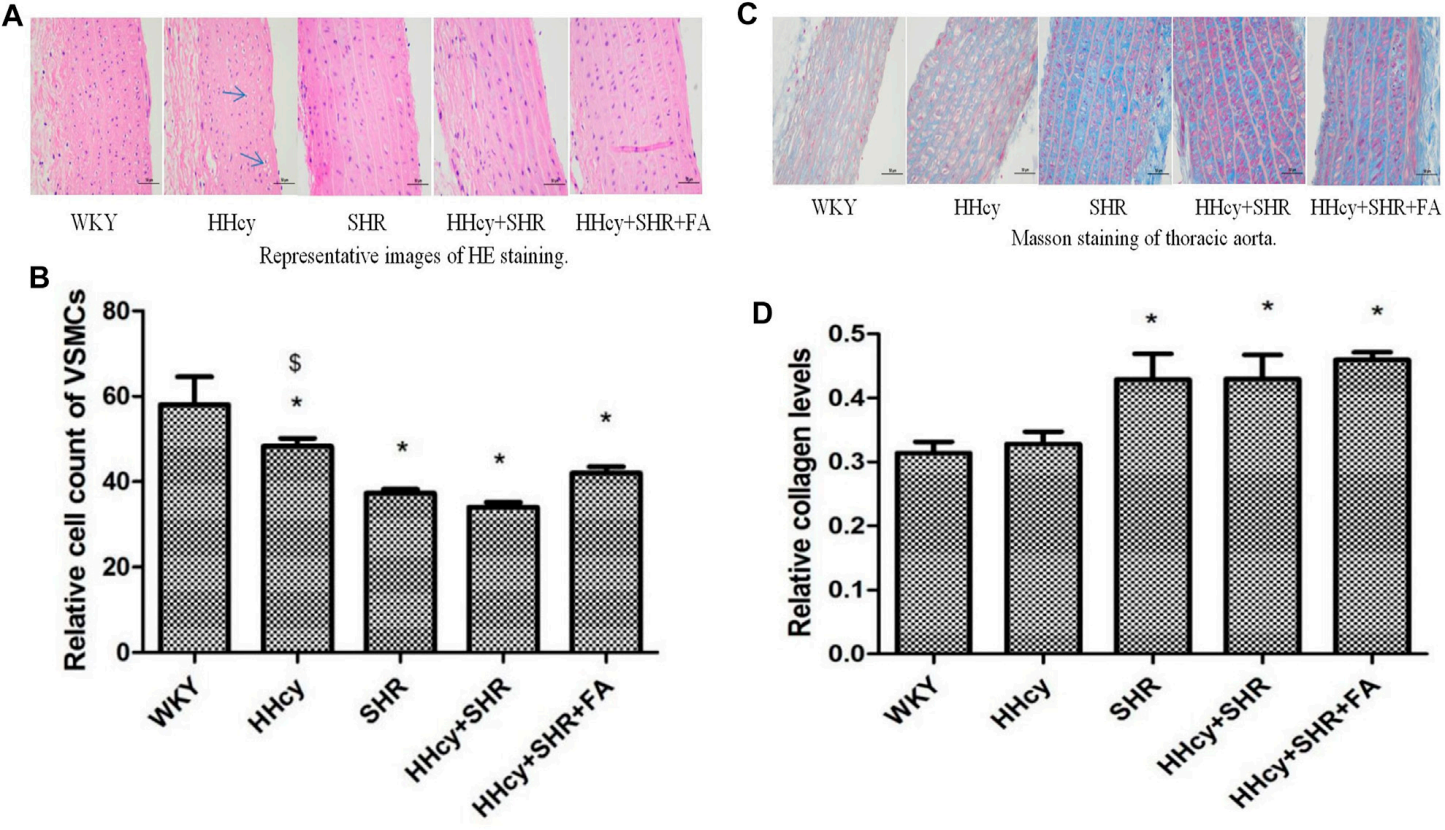
Hematoxylin and eosin (HE) and masson staining of aorta in Wistar-Kyoto (WKY) group, hyperhomocysteinemia (HHcy) group, spontaneously hypertensive rat (SHR) group, HHcy + SHR group, and HHcy + SHR + folate (FA) group. **(A)** Representative images of HE staining of rat aorta (original magnification, ×400). **(B)** Relative cell count of vascular smooth muscle cells (VSMC). **(C)** Representative images of masson staining of rat aorta (original magnification, ×400). **(D)** Semi-quantitative analysis of collagen deposition. The data are presented as the mean ± SD (*p < 0.05 vs. the WKY group, ^$^ *p* < 0.05 vs. the SHR group, ^&^ *p* < 0.05 vs. the HHcy + SHR group).

The images of HE staining (Figure 3A) showed that the arterial wall of all SHRs was significantly thicker than that of all WKYs. The arrow indicated more VMSCs degeneration and cytoplasmic vacuolation in the HHcy group.

The relative number of VSMCs in the vascular medium was measured by a computer microscope system at the same magnification. Due to the proliferation and hypertrophy of VSMCs and the deposition of extracellular matrix collagen in the pathological groups, the number of VSMCs in the same area under the same magnification was significantly reduced. Therefore, our data showed that compared with the WKY group, the number of VSMCs dropped the most in the HHcy + SHR group (*p* < 0.05), followed by the SHR group (*p* < 0.05) and the HHcy + SHR + FA group (*p* = 0.002). However, there was no statistical difference between the HHcy + SHR + FA group and the HHcy + SHR group (*p* = 0.064). (Figure 3B).

Collagen deposition, as another feature of vascular remodeling, was semi-quantified by Masson staining. Representative images of masson staining of rat aorta are in Figure 3C. Our data showed that collagen deposition in the aorta increased markedly in the HHcy + SHR group (*p* = 0.017), the SHR group (*p* = 0.018), and the HHcy + SHR + FA group (*p* = 0.003) compared with the WKY group. However, there was also no statistical difference between the HHcy + SHR + FA group and the HHcy + SHR group (*p* = 0.398) (Figure 3D).

In short, hypertension combined with HHcy significantly induced vascular structural changes, including VSMC proliferation and hypertrophy and extracellular matrix collagen deposition. Among them, hypertension was the main contributor. However, folate did not significantly reverse the changes in vascular structure.

### Comparison on Immune/Inflammation Indicators in Aortic Tissue

**FIGURE 4:**
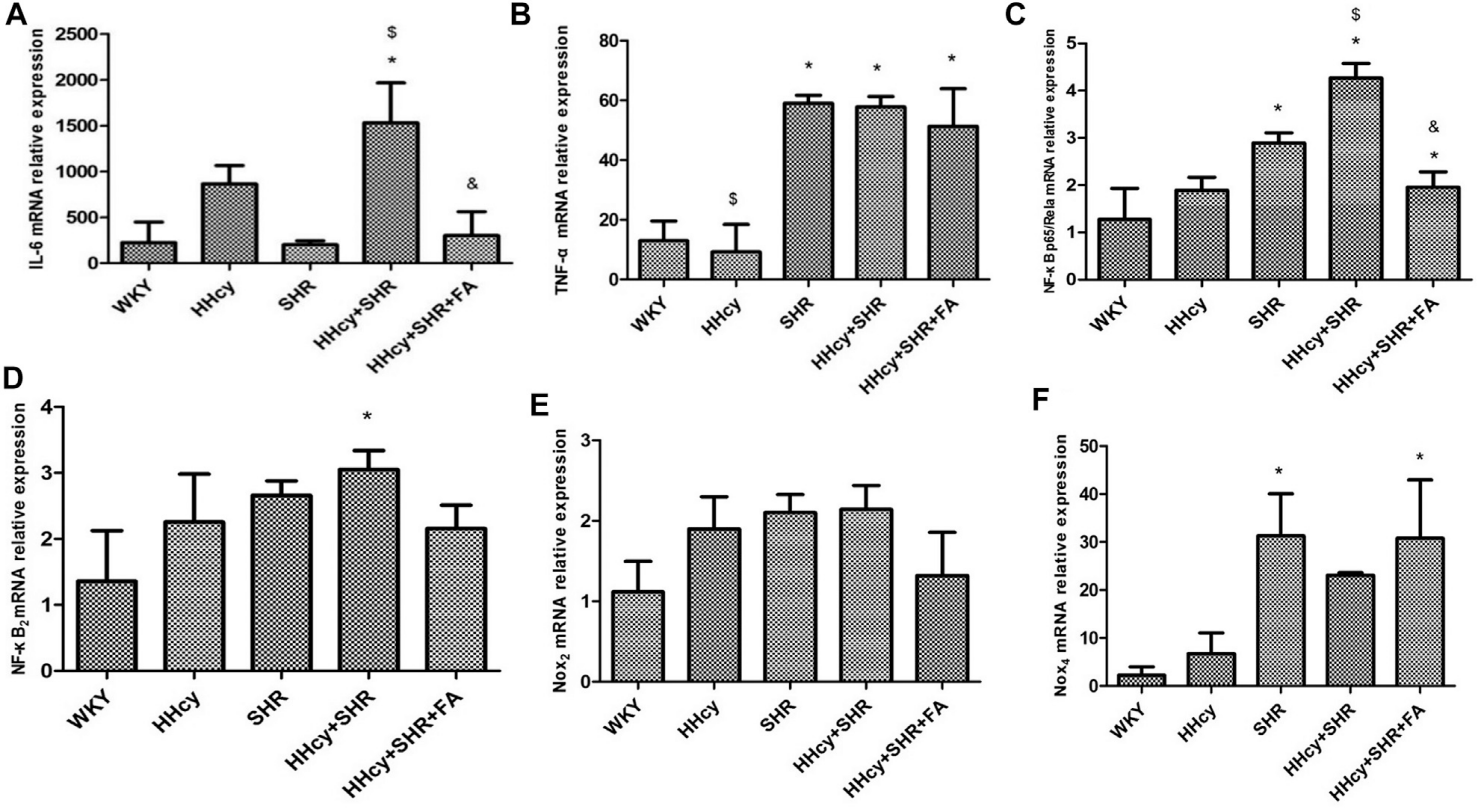
The mRNA relative expression levels by quantitative real-time polymerase chain reaction (qRT-PCR) analysis in Wistar-Kyoto (WKY) group, hyperhomocysteinemia (HHcy) group, spontaneously hypertensive rat (SHR) group, HHcy + SHR group, and HHcy + SHR + folate (FA) group. **(A)** The mRNA relative level of interleukin-6 (IL-6). **(B)** The mRNA relative level of tumour necrosis factor-alpha (TNF-α). **(C)** The mRNA relative level of nuclear factor-κ-gene binding (NF-κB) p65/Rela. **(D)** The mRNA relative level of NF-κB2. **(E)** The mRNA relative level of nicotinamide adenine dinucleotide phosphate (NADPH) oxidase (Nox)_2_. **(F)** The mRNA relative level of Nox_4_. Values represent means ± SD (*p < 0.05 vs. the WKY group, ^$^ *p* < 0.05 vs. the SHR group, ^&^ *p* < 0.05 vs. the HHcy + SHR group, n = 8).

**FIGURE 5:**
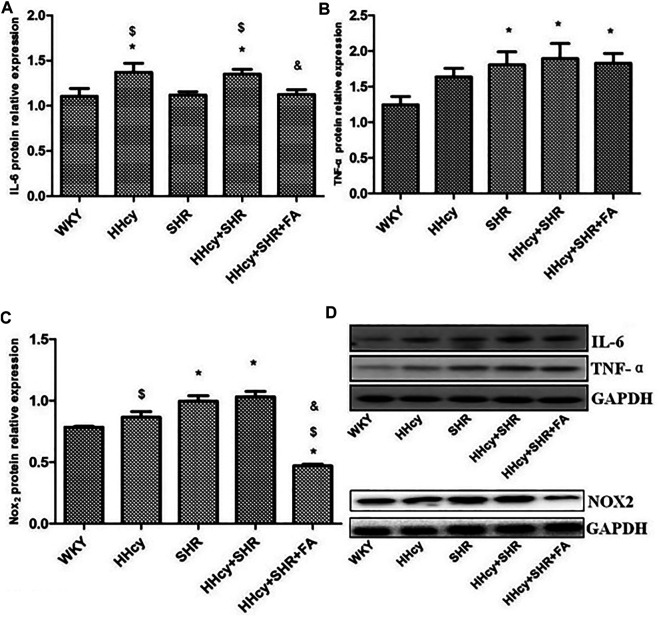
Protein quantitative analysis after normalized to GAPDH and representative western blotting (WB) images in Wistar-Kyoto (WKY) group, hyperhomocysteinemia (HHcy) group, spontaneously hypertensive rat (SHR) group, HHcy + SHR group, and HHcy + SHR + folate (FA) group. **(A)** The relative protein expression level of interleukin-6 (IL-6). **(B)** The relative protein expression level of tumour necrosis factor-alpha (TNF-α). **(C)** The relative protein expression level of nicotinamide adenine dinucleotide phosphate (NADPH) oxidase (Nox)_2_. **(D)** Representative WB images of IL-6, TNF-α, NOX_2_ and GAPDH. Values represent means ± SD (*p < 0.05 vs. the WKY group, ^$^ *p* < 0.05 vs. the SHR group, ^&^ *p* < 0.05 vs. the HHcy + SHR group, n = 8).

The aorta mRNA relative expression levels of IL-6, TNF-α, NF-κB p65/Rela and NF-κB2 were significantly increased in the hypertension combined with HHcy group compared with the WKY group (*p* < 0.05) (Figures 4A–D). What’s more, the aorta protein relative expression levels of IL-6 and TNF-α were also significantly increased in the hypertension combined with HHcy group compared with the WKY group (*p* < 0.05) (Figures 5A,B). Furthermore, the protein relative expression levels of IL-6 were increased in the HHcy group than the SHR group (*p* = 0.029; Figure 5A), whereas, the mRNA relative expression levels of TNF-α were increased in the SHR group than the HHcy group (*p* = 0.001; Figure 4B). After folate intervention, the aorta mRNA expression levels of IL-6 and NF-κB p65/Rela were significantly decreased in the HHcy + SHR + FA group than the HHcy + SHR group (*p* < 0.05) (Figures 4A,C), and the aorta protein expression levels of IL-6 were also significantly decreased in the HHcy + SHR + FA group than the HHcy + SHR group (*p* < 0.05) (Figure 5A).

In summary, both HHcy and hypertension were related to rat arterial inflammation, and HHcy combined with hypertension could significantly increase arterial inflammation. Conversely, folate showed a significant inhibitory effect on the immune/inflammation caused by hypertension combined with HHcy.

### Comparison on Oxidative Stress Indicators in Aortic Tissue

The aorta mRNA relative expression levels of Nox2 and Nox4 were only an increasing trend in the HHcy + SHR group compared with the WKY group (*p* > 0.05), and there was no significant change between the HHcy + SHR + FA group and the HHcy + SHR group (*p* > 0.05) (Figures 4E,F). However, the protein relative expression level of Nox2 was significantly increased in the HHcy + SHR group (*p* = 0.001) and the SHR group (*p* = 0.002) compared with the WKY group. Additionally, the protein relative expression level of Nox2 was significantly decreased in the HHcy + SHR + FA group than the HHcy + SHR group (*p* < 0.05; Figure 5C).

In summary, the HHcy + SHR group rats highly expressed Nox2 protein molecule, and folate showed a positive effect on reducing Nox2 protein expression. However, the difference in the mRNA relative expression of Nox2 was not as obvious as that in the protein expression, which needs further verification.

## Discussion

Our study found that the Hcy level of SHR was significantly higher than that of WKY. It indicates that hypertension may have metabolic abnormalities, especially when hypertension is accompanied by HHcy that is the accumulation of Hcy under an abnormal metabolic state. Therefore, in a sense, hypertension combined with HHcy is related to metabolic-related diseases. Furthermore, our results demonstrated that hypertension combined with HHcy had the most significant arterial pathological changes, and the overall expression levels of oxidative stress and immune/inflammation molecules in the arterial tissue were also the most significant. Moreover, hypertension is the main contributor for the pathological changes of the arteries in the hypertension combined with HHcy rats, and hypertension itself is more important than HHcy in inducing oxidative stress and immune/inflammation to cause arterial damage.

Epidemiological studies have suggested that HHcy is associated with increased risk of vascular disease (Boushey, 1995; Graham et al., 1997). Studies have proposed that HHcy causes atherosclerosis by oxidative stress and immune inflammation (Koch et al., 1998; Charalambos et al., 2006). However, our experimental results showed that HHcy alone did not induce rat arterial remodeling. Although HHcy increased the levels of oxidative stress in the circulation, it did not induce the high expression of NOX2 and NOX4 in rat arterial tissue. Only after cooperating with hypertension, HHcy increased the expression levels of IL-6 and NF-κBp65/Rela. Furthermore, studies have suggested that the inflammatory response induced by HHcy is related to Hcy-protein modification (Jakubowski, 2019). Hcy-protein changes the normal structure and function of the protein and even acts as an abnormal antigen to trigger an immune/inflammatory response. Together these findings suggest that HHcy promotes an inflammatory status and can synergistically aggravate the arterial damage of hypertension, at least in part, through the NF-κB p65/Rela/IL-6 molecular pathway.

Folate participates in an essential one-carbon metabolism in the body and affects hyper-methylation patterns of genetic material and/or metabolic molecules (Stenvinkel et al., 2010). In addition, folate provides one-carbon molecule for Hcy to promote the reverse synthesis of methionine (Henrieta et al., 2016) and then significantly reduces the concentration of Hcy. A study found that low folate concentration, independent of HHcy, may promote atherogenesis (Durga et al., 2005), not only through a decrease of antioxidant capacity and endothelial function but also through exacerbation of inflammation and adhesion of inflammatory molecules in the vessel wall (Li et al., 2006). Whereas, supplementing vegetables and fruits rich in folate can reduce inflammation and oxidative stress (Holt et al., 2009). Our research showed that folate significantly reduced the levels of HHcy and MDA (oxidative stress product) and significantly increased the level of SOD (antioxidant stress molecule). Moreover, the SOD activity level in the HHcy + SHR + FA group even exceeded the SHR group. However, folate did not significantly reduce the gene expression of NOX2 and NOX4. Therefore, we infer that folate has a strong anti-oxidative stress ability, not by inhibiting or at least not by significantly inhibiting NOX2 and NOX4, but by directly stimulating the activity of antioxidant molecule, for example, nuclear factor erythroid 2-related factor 2 (Nrf-2)/heme oxygenase-1 (HO-1)/SOD pathway molecules. The Nrf-2/HO-1/SOD is an important antioxidative stress pathway (Fujiki et al., 2019), and another study by our experimental team has confirmed that folate can increase the expression level of Nrf-2/HO-1/SOD.

We also discovered that folate significantly reduced the expression levels of IL-6 and NF-κB p65/Rela, but not TNF-α level. Moreover, the reduction in NF-κB p65/Rela/IL-6 level was in parallel with the impact of folate on HHcy level. It is worth noting that both IL-6 and TNF-α were increased in hypertension combined with HHcy rats, among which HHcy was the contributor for increased IL-6, while hypertension was the main factor for increased TNF-α. Therefore, we speculate that folate reverses the NF-κB p65/Rela/IL-6 induced by HHcy. So, does folate reduce inflammation indirectly by inhibiting HHcy, or does it have anti-inflammatory properties itself? In order to explore this issue, we reviewed and summarized the related literature on folate and immune/inflammation, focusing on hypertension and CVD.

Literature results show that the role of folate in anti-inflammation and immune regulation is controversial. Some studies believed that folate has anti-inflammation and immune regulation effects. For example, Lei et al. (2019) found that folate can serve as a potential therapeutic agent against vascular disease through potential suppression on angiogenesis, inflammation and oxidative stress. Ahmed et al. (2018) found that folate protects against prenatal nicotine -induced cardiac injury by decreasing serum TNF and cyclooxygenase-2 (COX-2) expression. Kemse et al. (2017) found that the combined supplementation of folate, vitamin B12, and omega-3 fatty acids decreases TNF-α level. Tousoulis et al. (2014) found that folate decreases IL-6 level. Solini et al. (2006) found that a short-term folate supplementation reduces the circulating levels of certain inflammatory mediators independently of weight changes, thus suggesting a potential therapeutic role for folate in preventing atherosclerosis and CVD. On the contrary, some studies believed that folate cannot change the inflammatory state of the body. For example, Christen et al. (2018) found that combined treatment with folate, vitamin B6 and vitamin B12 lowers HHcy concentration, but it does not alter major biomarkers of vascular inflammation. Mierzecki et al. (2014) found that folate supplementation has no influence on the coagulation, inflammatory and lipid parameters in subjects with atherosclerosis risk factors. Bleie et al. (2010) found that in patients with stable coronary atherosclerotic heart disease (CAD), HHcy-lowering therapy with vitamin B (including folate) does not affect the levels of inflammatory markers associated with atherogenesis. Mangoni (2006) summarized epidemiological studies and interventional studies and then concluded that folate does not significantly change inflammatory markers. Besides the above arguments, there are some viewpoints that folate indirectly leads to a decrease in inflammation by reducing the concentration of HHcy (Baszczuk et al., 2015) or by intervening in the one-carbon cycle (Kemse et al., 2014).

In short, some literature indicate that folate does not significantly change the level of inflammatory markers of CVD, which may be related to the inherent etiology of CVD that may trigger a unique immune inflammatory response. Furthermore, folate can neither eliminate the inherent etiology of CVD nor completely block its pathogenesis. This is why folate fortification is unsuccessfully tested in humans with established CVD. Although there are more studies supporting the potential anti-atherosclerosis and anti-inflammatory effects of folate, the current evidence is still limited. Because the research results of folate in preventing CVD are ambiguous, it seems necessary to conduct further research, which will explain in which cases folate supplementation is useful. However, we can at least confirm that folate indeed reverse the inflammatory response which is caused by HHcy, regardless of whether folate indirectly inhibits the NF-κB p65/Rela/IL-6 pathway by reducing HHcy or whether it has direct anti-inflammation and immune regulation effects. Our findings may provide additional explanations and further insights into the arterial protection mechanism of folate. Therefore, folate will gain more attention because of its potential to weaken arterial damage factors in hypertension combined with HHcy.

## Conclusion

HHcy synergistically aggravated the arterial damage factor of hypertension through NF-κB p65/Rela/IL-6 signaling pathway, which could be the target of folate against immune/inflammation. Additionally, folate also exhibited powerful antioxidant properties.

## Data Availability

The raw data supporting the conclusions of this article will be made available by the authors, without undue reservation.
